# Behavioural risks in female dogs with minimal lifetime exposure to gonadal hormones

**DOI:** 10.1371/journal.pone.0223709

**Published:** 2019-12-05

**Authors:** Melissa Starling, Anne Fawcett, Bethany Wilson, James Serpell, Paul McGreevy

**Affiliations:** 1 Sydney School of Veterinary Science, Faculty of Science, University of Sydney, Sydney, New South Wales, Australia; 2 School of Veterinary Medicine, University of Pennsylvania, Philadelphia, Pennsylvania, United States of America; Tokat Gaziosmanpasa University, TURKEY

## Abstract

Spaying of female dogs is a widespread practice, performed primarily for population control. While the consequences of early spaying for health are still being debated, the consequences for behaviour are believed to be negligible. The current study focused on the reported behaviour of 8981 female dogs spayed before 520 weeks (ten years) of life for reasons other than behavioural management, and calculated their percentage lifetime exposure to gonadal hormones (PLGH) as a proportion of their age at the time of being reported to the online Canine Behavioral Assessment and Research Questionnaire (C-BARQ). We found that 23 behaviours differed between entire and spayed dogs, of which 12 were associated with PLGH and 5 with age-at-spay (AAS). Two behaviours, chewing and howling, were significantly more likely in dogs with longer PLGH. In contrast, longer PLGH was associated with significantly reduced reporting of 10 (mostly unwelcome) behaviours. Of these, one related to fearfulness and three to aggression. The current data suggest that dogs’ tendency to show numerous behaviours can be influenced by the timing of spaying. They indicate how female dog behaviour matures when gonadal hormones are allowed to have their effect. The differences reported here between undesirable behaviours of spayed and entire dogs were in the range of 5.33% and 7.22%, suggesting that, for some dogs, partial or complete denial of maturation may reduce howling and chewing and improve retrieval and recall, but have other undesirable consequences. Veterinarians may take these data into account to discuss the risks and benefits of spaying with clients, and the timing of the procedure.

## Introduction

Domestic dogs (*Canis lupus familiaris*) are extremely popular globally as companion animals: approximately 38 per cent of Australian households owns a dog [1], compared with 26 per cent of households in the UK [2], and 60.2 million dog-owning households in the USA [3]. Therefore, meeting the behavioural needs of this substantial population of animals has a potentially huge impact on animal welfare. Unwelcome dog behaviours that jeopardize human health and animal welfare arise largely because of fear, frustration and thwarted motivation, when carers fail to meet dogs’ needs or prepare dogs adequately for life in human households and settlements[4].

Dog behaviour may differ between sexes, but the factors driving these differences may be poorly understood. For example, male dogs tend to predominate in bite statistics [5–9]. It has been suggested that this higher incidence of aggression is a reflection of the relative boldness of male dogs when compared with female dogs. Boldness is a so-called super-trait that manifests with increased engagement in social interactions and a generalised lack of fear [10,11]. Entire (also known as intact) dogs of both sexes are generally bolder than their gonadectomised (also known as neutered or desexed) counterparts [10]. This has led to recommendations that gonadectomy of male dogs may reduce problem behaviours or reduce dog-bites in the community [12]. Recent studies have found no difference in aggression in neutered male dogs compared to entire male dogs [13–17].

The prevalence of gonadectomy of dogs varies across the globe. For example, in Australia, there is evidence that 85% of female dogs and 77% of male dogs are gonadectomised [18] while in the U.S., an estimated 83% of all dogs are gonadectomised [19]. In contrast, gonadectomy rates remain low in some countries. For example, among dogs attending UK veterinary clinics (n = 148,741), only 41.1% were gonadectomised [20], although a more recent survey of the records of 143 practices encompassing 329 clinics in Great Britain found that 57.1% of dogs were gonadectomised [21]. In a sample of dogs (n = 10,519) owned by German-speaking dog enthusiasts, 43.1% were gonadectomised [22].

The reasons for gonadectomy in dogs vary, but the procedure is most commonly encouraged as a means of population control; historically being conducted at, or just before, sexual maturity. Gonadectomy has also been recommended in some countries as a means of preventing medical and behavioural problems [23,24]. In recent times, the benefits of early-age gonadectomy (at less than 12 weeks of age) for population control have been emphasised [25]. In addition, early-age gonadectomy facilitates early adoption in those shelters with a policy of only allowing adoption of gonadectomised animals, potentially reducing length of stay (LOS), as younger animals would not have to be retained until they have reached sexual maturity in order to be gonadectomised before adoption.

Thousands of dogs are euthanased in shelters and pounds annually in many developed countries [26–29]. The reasons why they are euthanised vary, but problem behaviours feature heavily [30]. Recent reports suggest unwelcome behaviours are more prevalent in dogs that have been gonadectomised (for reasons other than behavioural problems) [31]. One possible reading of these data is that gonadectomy may reduce the numbers of unwanted dogs but may also increase the likelihood of problem behaviours that reduce the appeal of the gonadectomised dogs, compromise the human-animal relationship, and make dogs more vulnerable to being surrendered. There is a need to clarify if this is the case so that gonadectomy of dogs is in the best interests of dogs.

Gonadectomy may have an impact on health and longevity. Such impacts may differ depending on the sex of the dog [32]. Increased investigation of the role of canine gonadectomy in disease incidence has raised concerns about blanket recommendations for gonadectomy in some countries [23,33]. In males, gonadectomy is associated with an increased risk of prostate cancer but eliminates the risk of testicular disease and many disorders that depend on androgens, such as prostatitis, benign prostatic hyperplasia, perineal hernias and perineal adenomas, as reviewed previously [34]. In females, ovariohysterectomy has been traditionally advocated to avoid the risk of pyometra (pus in the uterus) which is a potentially life-threatening bacterial infection associated with severe complications including sepsis, septic shock, peritonitis, systemic bacterial infection and multi-organ failure [35]. The pyometra-related mortality rate was 10 per cent when euthanasia was included, and 1 per cent in treated dogs [36]. With the exception of stump pyometra, a rare infection which typically occurs due to residual hormone-producing ovarian remnants [37], gonadectomy prevents this condition. In countries where elective spaying is rare, pyometra is common. For example, in Sweden, the rate was 23–24% of dogs under the age of ten years [38]. In contexts where spaying is common, a reduction in the rate of spaying is associated with increased rates of pyometra. For example, in a retrospective study of five RSPCA hospitals in the UK over a 5-year period, the overall prevalence of pyometra was 2.2%, but incidence increased as the rate of elective ovariohysterectomy declined [39]. However, pyometra does not typically develop without the influence of progesterone [40,41], meaning that a hysterectomy alone or an ovariectomy alone should be sufficient to avoid pyometra. It has also been shown that the risk of developing pyometra is not higher in dogs that have had an ovariectomy than those with an ovariohysterectomy [42]. Accordingly, ovariectomy is becoming the standard in Europe [42]. Other health consequences of gonadectomy may be less direct. For example, spayed female golden retrievers had a 3–4 fold increase in the rate of some cancers [23]. Similarly, studies of golden retrievers [24] and German shepherd dogs [25] indicate that early neutering can significantly predispose dogs to joint disorders, including hip dysplasia.

In females, spaying was recommended as a means of reducing the risk of mammary neoplasia [43] but a recent systematic review described the evidence for this association as being only weak [44]. However, it has been argued that this finding is a reflection of the veterinary evidence base in general, rather than an indictment of conclusions based on multiple studies investigating the relationship between gonadectomy and mammary tumours [33]. Other physical and behavioural consequences are outlined in Table 1. It should be noted that the associations between neutering/gonadectomy and disorders described in Table 1 are not necessarily causal and that many of these outcomes are contested. An abiding issue is that some studies compare early neutering with standard age neutering, and some studies compare neutering with not neutering. There is a lot of conflation of these two very different comparisons in discussions which confuses the debate. In addition, it is possible that potential confounders, such as lifestyle, and breed predispositions to disorders, such as obesity, diabetes and urinary incontinence, are poorly addressed among many studies, especially the earlier ones. While a comprehensive review of this literature is beyond the scope of this paper, it is important to note that the benefits and risks of surgical neutering may vary according to individual factors, including breed. For example, the risk of development of urethral sphincter mechanism incompetence after surgical neutering may vary with the size of the dog [45].

**Table 1:** Reported potential physical and behavioural benefits versus disadvantages of neutering female dogs.

	Benefits	Disadvantages
Physical	Prevention of pregnancy and related disorders including pregnancy toxaemia, diabetes mellitus, pregnancy associated pyelonephritis, uterine torsion, uterine rupture, dystocia, mastitis, pseudopregnancy [46–48]	Surgical complications (6.1–27%) [25,53]
	Decrease in incidence of vaginal prolapse [33,47]	Anaesthetic complications [54,55]
	Decreased risk of sexually transmitted infection (e.g. *Brucella canis*)[49]	Pseudopregnancy (less commonly than in intact dogs) [48]
	Prevention of ovarian and uterine neoplasia [33]	Potential for stump pyometra if ovarian tissue retained/progesterone administered [56]
	Prevention of other reproductive tumours (for example vaginal leiomyomas)	Increased incidence of urinary incontinence [44,51,57]
	Prevention of pyometra (normal incidence 19–25% in dogs by the time they reach ten years old) [36,39] and associated complications including surgical and anaesthetic complications [50]	Increased risk of urinary tract infection [58]
	Decrease in incidence of mammary neoplasia (normal incidence 3.4–13%) [25,36,51]	Atrophic vaginitis [59]
	Increased lifespan [33,52]	Perivulvular dermatitis [59]
		Increased risk of rupture of cranial cruciate ligament [51,60,61]
		Decreased caloric requirements with increased risk of obesity[62]
		Increased incidence of some neoplastic disease (for example, cardiac tumours, osteosarcoma, splenic haemangiosarcoma, cardiac haemangiosarcoma, hyperadrenocorticism, mast cell tumours, lymphoma, transitional cell carcinomas) [60], [61]
		Increased risk of epilepsy [60].
		Increased risk of intervertebral disc disease [60].
Behavioural	Decrease in oestrus-associated behaviours [25]	Increased possessive aggression [63]
	Reduced risk of vehicular injury[60]	Increased owner-directed aggression [64]
		Increased reactivity to approach by stranger (German shepherd dogs spayed between 5 and 10 months of age) [65]
		Increased fearful behaviour in Labradors in response to loud noise, unfamiliar objects approaching on or near the sidewalk, and approach by unknown dog that is barking, growling or jumping [66]. (Fearfulness of approach by unknown dog that is barking, growling or jumping was more pronounced in dogs spayed after puberty.)
Other	In some jurisdictions, reduced dog registration fees	
	Owner convenience	

Despite recommendations regarding gonadectomy and aggression in dogs, the effects of gonadectomy on dog behaviour have rarely been subjected to detailed inquiry. If gonadectomy increases the risks of certain behaviours, it may have a negative influence on the human-animal bond [67]. Recent studies have identified some concerning and surprising trends. For example, gonadectomised dogs of both sexes have been reported to be marginally more prone to aggression towards unfamiliar humans than entire dogs [24]. Weight gain and related lethargy are seen in many gonadectomised dogs [68], and a metanalysis of studies designed to determine the maintenance energy requirements of dogs found that maintenance energy requirements were much less in neutered dogs [62]. Owners should be counselled to avoid these outcomes [69] e.g., by feeding low-energy diets. This concern is reflected in the availability of commercial diets specifically designed for neutered dogs. Unfortunately, energy-restriction may increase the perceived value of rations to gonadectomised dogs, potentially leading to food-related aggression (resource guarding) [69]. As it happens, increased appetite and human-directed aggression have been reported in spayed bitches [70]. In this study, the risk was found to be greatest in puppies under twelve months old at the age of neutering, who were already displaying aggression. Reports from owners of 209 male and 382 female dogs revealed that, after gonadectomy, male dogs more often showed distinct behavioural changes than female dogs [68]. For 49 of 80 (61%) aggressive male dogs and 25 of 47 (53%) female dogs were reported to be more gentle after gonadectomy [71]. However, 10 females were described as becoming aggressive after gonadectomy, and voluntary food intake increased in 32% of female dogs. Gonadectomy may also have a negative impact on the learning ability of dogs. Tests of spatial learning, memory and reversal learning tasks using a T-maze showed that 81% of entire females successfully completed the whole procedure compared to 56% of spayed females [71,72]. It has also been reported that spayed female dogs are more likely to prefer egocentric navigational strategies rather than allocentric [73], and that spayed females take longer to respond to the social cue of pointing from a human and are more likely to respond incorrectly than entire female dogs [74].

Recent studies of male dogs have revealed behavioural risks associated with minimal lifetime exposure to gonadal hormones that may offset some population-control benefits of gonadectomy [24,31]. The current study examines data from the owners of female dogs to explore relationships between various canine behavioural attributes, as reported through C-BARQ, and the timing of spaying considered as both age-at-spay (AAS) and percentage lifetime exposure to gonadal hormones (PLGH).

## Method

### C-BARQ

The C-BARQ is an owner-completed survey instrument designed to provide quantitative assessments of a wide array of behavioural characteristics of dogs. It consists of 100 items that ask respondents to indicate, using a series of 5-point ordinal rating scales, their dogs’ typical responses to a variety of everyday situations during the recent past. The scales rate either the intensity (aggression, fear and excitability subscales) or frequency (all remaining subscales and miscellaneous items) of the behaviours, with a score of 0 indicating the absence of the behaviour and a score of 4 indicating the most intense or frequent form of the behaviour [75,76]. The online C-BARQ survey (http://vetapps.vet.upenn.edu/cbarq/) was reviewed by the University of Pennsylvania Institutional Review Board and determined to be exempt from IRB approval because no personal identifying information other than email address is collected from dog owners during survey completion. The requirement for informed consent was also waived. Participants are not actively recruited to complete the C-BARQ. Beginning in 2006, the original online survey was advertised via an article in the newsmagazine of the School of Veterinary Medicine at the University of Pennsylvania (http://www.vet.upenn.edu/bellwether/v64/article10.shtml), and by notices sent to Philadelphia-area veterinary clinics and the top 20 USA breed clubs based on AKC registrations. Since that time, information about the survey has been disseminated via word-of-mouth and social media among dog owners who visit the website and complete the questionnaire of their own volition.

The C-BARQ also asks respondents to specify the reasons for spaying. For the purposes of the current study, we drew data only on female dogs spayed before 520 weeks (10 years) of age. We accepted data on dogs spayed up to two weeks prior to C-BARQ evaluation for the following reasons: required by breeder/shelter (n = 3163), birth control (n = 4518), prevention of health problems (n = 1084), and correction of health problems (n = 216). We excluded dogs (n = 1552) spayed for the following reasons: to correct a behaviour problem (n = 86), to prevent a behaviour problem (n = 100), recommended by vet (n = 552) and unknown (n = 814).

### Candidate attribute selection

Because of the large numbers of attributes involved, candidate attributes selection was undertaken prior to the primary analysis, to identify attributes in which there were clinically significant behavioural differences between entire and gonadectomised dogs. C-BARQ scores from 3844 entire female dogs and 11560 spayed female dogs were compared

Each behaviour was coded as a binary variable (high/low) as per McGreevy et al. [31], according to the score received in the C-BARQ and behaviours were then compared between spayed and intact female dogs. For most studied behaviours, dogs were considered to have demonstrated either a lower tendency for the behaviour (a score of Never) or a higher tendency for the behaviour (any other score). For behaviours “Playful, puppyish and boisterous” and “Active, energetic, always on the go”, a more moderate division of scores was applied where scores of Never, Seldom or Sometimes were considered a low tendency, and scores of Usually or Always were considered a high tendency. The more moderate division was intended to reflect the presumed desirability of moderate frequency of these behaviours. Several Trainability behaviours (“When off the leash, returns immediately when called”, “Obeys the ‘sit’ command immediately”, “Obeys the ‘stay’ command immediately”, “Seems to attend/listen closely to everything you say or do” and “Will ‘fetch’ or attempt to fetch sticks, balls, or objects”) were also classified differently, with Usually or Always being grouped together to represent a high tendency. As discussed by McGreevy et al. [31], ‘Always’ was subjectively considered to be too stringent a standard to be truly informative for these behaviours (i.e., a dog that ‘Usually’ sits immediately on command would be reasonably considered to have a high tendency to obey the sit command).

The percentage of entire and spayed dogs displaying a high tendency for the behaviour for each attribute were compared. Of the 100 behaviours, 23 showed a difference of more than 5% in the proportion of higher tendency dogs between the “entire” and the “spayed” cohorts.

#### PLGH

The age of the dog when it was evaluated through C-BARQ and its reported AAS were used to calculate its lifetime exposure to gonadal hormones at the time of C-BARQ evaluation using the formula: AAS/Age at time of C-BARQ evaluation x 100. It is worth noting that the PLGH does not claim to reflect on the full life of the dogs that are yet to die, only to describe the proportion of its lifetime, at the time of C-BARQ evaluation, that it has had gonadal hormones circulating. Clearly, AAS and PLGH are related but the latter indicates how long the dog has lived with gonadal hormones until the age at which she was spayed and can reveal the influence of gonadal hormones on behaviour after puberty, reflecting the role of circulating hormones in the normal behavioural biology of female dogs.

It is worth noting that we have no behavioural data on the dogs’ entire lifespan. We combined C-BARQ responses of 3 and 4 on the severity of a behaviour scale to arrive at four gradations: 0, 1, 2, 3+. As stated above, we nominated a 5% difference in the frequency of behaviours between spayed and entire dogs as the threshold for a trait to undergo further analysis.

### Statistical analysis

The C-BARQ data included ordinal item-scores for 100 behavioural attributes [75]. Because linear models make assumptions based on a continuously varying underlying trait, rather than a composite quasi-continuous trait assembled from ordinal scorings, analysis was undertaken on the ordinal scores directly using models better suited to ordinal data.

#### Model age of spaying

The effect of AAS on C-BARQ scores was evaluated by a cumulative link model [77] with a logit link function, using the polr() function of the MASS package of R statistical software (R Foundation for Statistical Computing, Vienna, Austria. URL https://www.R-project.org/).

This ordinal logistic regression model had the following terms:

Y ~ AAS + AgeAtEvaluation + Breed + Weight + Breed.Weight + FirstOwned + OtherDogs + Roles.

where y denotes the ordinal C-BARQ score and logit link function, AAS (weeks), Breed of dog (for the 29 breeds and crosses effects tested, *see* S1 Table), Weight is the weight of the dog (in pounds reported at C-BARQ evaluation), Breed.Weight is an interaction term between breed and weight, AgeAtEvaluation denotes the age of the dog at C-BARQ evaluation (in weeks), FirstOwned denotes a binary variable indicating whether or not this is the owner’s first dog, Other Dogs is a binary variable indicating whether or not there are other dogs in the household, and Roles is a categorical variable indicating whether the dog is reported to be involved with breeding/showing, working roles, field trials/hunting, other sports, or none of the above.

#### Models PLGH evaluation

We used two candidate models:

*a full model*,

Y ~ PLGH + AgeAtEvaluation + PLGH.AgeAtEvaluation + Breed + Weight + Breed.Weight + FirstOwned + OtherDogs + Roles,

which includes PLGH and an interaction term between PLGH and AgeAtEvaluation; and

*a reduced model*,

Y ~ PLGH + AgeAtEvaluation + Breed + Weight + Breed.Weight + FirstOwned + OtherDogs + Roles,

which does not include this second interaction term.

After calculation of the models, the Anova() function of the “car” package for R was used to calculate Anova tables for the model effects. A Holm-Bonferroni correction for multiple comparisons was applied to the *P* values arising from models for each trait to further control the risk of Type 1 error resulting from the large number of traits (i.e. 23) being tested. Once a Holm-Bonferroni correction for multiple comparisons had been applied, the interaction of PLGH and AgeAtEvaluation was significant for only 7 of the 23 traits of interest (chewing inappropriate objects, aggression when approached directly by an unfamiliar male dog while being walked/exercised on a leash., pulling the leash, showing aggression when barked, growled, or lunged at by another (unfamiliar) dog, (Recall, Fetching objects, and Rolling in odours). Therefore, the reduced model was preferred, and its results are discussed further.

## Results

Among the 8981 dogs spayed prior to 520 weeks of age, AAS was positively skewed with a median of 30 weeks (6.9 months), a mean of 62.9 weeks (14.5 months) and a standard deviation of 78.1 weeks (18.0 months). PLGH for these dogs was less positively skewed, with a median of 21.2%, a mean of 31.3% and a standard deviation of 27.2%.

Of the 23 behaviours that differed between entire and spayed dogs (see S2 Table), 12 were associated with PLGH and 5 with AAS. Specifically, 10 behaviours were significantly reduced for either AAS or PLGH or both

Table 2 shows that the fear-related behaviours that decreased with increasing PLGH included only when barked, growled or lunged at by an unfamiliar dog, odds ratio of 0.997 (with an uncorrected 95% confidence interval of 0.996–0.999). This odds ratio indicates that for every additional 1% of her life before C-BARQ evaluation that passes before she is spayed the odds of a dog having a higher score for this behaviour is multiplied by 0.997(CI = 0.996–0.999). While appearing small (i.e. close to 1) on a weekly basis, this is equivalent to odds multiplied by 0.963(CI = 0.947–0.979) for an increased PLGH of 10%, (about a 5-month delay in spaying for a dog evaluated by C-BARQ at 4 years of age).

**Table 2:** Association of timing of spaying (AAS and PLGH) with fear- and anxiety-related responses for female dogs.

Response		ANOVA (type II) statistics		
	Age At Spay (AAS Model) likelihood-ratio χ^2^	*P* value	PLGH (Reduced PLGH Model) Likelihood -ratio χ^2^	*P* value
When having his/her feet towelled by a member of the household	0.388	0.534	0.984	0.321
When barked, growled, or lunged at by an unfamiliar dog	8.128	0.004	20.473	<0.001*
In response to sudden or loud noises (e.g. vacuum cleaner, car backfire, road drills, objects being dropped, etc.)	0.102	0.749	0.149	0.699
When examined/treated by a veterinarian	0.302	0.583	7.835	0.005
During thunderstorms, firework displays, or similar events	0.326	0.568	0.725	0.394
When first exposed to unfamiliar situations (e.g. first car trip, first time in elevator, first visit to veterinarian, etc.)	1.496	0.221	0.123	0.726
In response to wind or wind-blown objects	2.342	0.126	5.298	0.021
When having nails clipped by a household member	3.592	0.058	7.52	0.006
When groomed or bathed by a household member	1.090	0.297	0.125	0.724

* Significant p values at the level 0.05 after Holm-Bonferroni correction

Table 3 shows the association of timing of spaying with the expression of aggressive behaviours, with a strong, statistically significant increase in the likelihood of aggression when delivery workers approach the home, (OR = 0.994,Uncorrected 95% Confidence interval of 0.9992–0.9998); when approached by an unfamiliar male dog while on leash (OR = 0.997, Uncorrected 95% Confidence interval of 0.995–0.999); and when being barked, growled or lunged at by an unfamiliar dog (OR = 0.996, Uncorrected 95% Confidence interval of 0.995–0.998). Aggression toward delivery workers was also associated with younger AAS (OR = 0.999, Uncorrected 95% Confidence interval = 0.998–0.999), but AAS was not significantly associated with the other aggressive behaviours.

**Table 3:** Association of timing of spaying with aggression-related responses for female dogs.

Type of aggression	ANOVA (type II) statistics			
	Age At Spay (AAS model) Likelihood-ratio χ^2^	*P* value	PLGH (ReducedPLGH Model) Likelihood-ratio χ^2^	*P* value
When mailmen or other delivery workers approach your home	19.350	<0.001*	51.21	<0.001*
When approached directly by an unfamiliar **male** dog while being walked/exercised on a leash	0.000	>0.999	11.561	0.001*
When barked, growled, or lunged at by another (unfamiliar) dog	0.050	0.832	11.3	0.001*

* Significant p values at the level 0.05 after Holm Bonferroni correction.

The effects of PLGH on the likelihood of owners reporting excitable behaviour and other miscellaneous problem behaviours is shown in Table 4, with the specific effects of PLGH shown in S2 Table. There was an increased likelihood of excitable behaviour when the doorbell rings, (OR = 0.989, Uncorrected 95% Confidence interval of 0.987–0.991)) and an increase in the likelihood of excitable behaviour before being taken on a car trip [(OR = 0.997, Uncorrected 95% Confidence interval of 0.995–0.998).

**Table 4:** Association of timing of spaying with excitability, energy and miscellaneous responses for female dogs.

Response	ANOVA (type II) statistics			
	AAS likelihood-ratio χ^2^	*P* value	PLGH (Reduced PLGH Model) Likelihood -ratio χ^2^	*P* value
**Excitability**				
When doorbell rings.	71.767	<0.001*	166.107	<0.001*
Just before being taken on a car trip.	5.149	0.023	18.508	<0.001*
**Trainability**				
When off the leash, returns immediately when called.	0.010	0.921	10.116	0.00147*
Easily distracted by interesting sights, sounds or smells.	0.049	0.826	0.186	0.666
Will ‘fetch’ or attempt to fetch sticks, balls, or objects.	68.100	<0.001*	64.39	<0.001*
**Separation**				
Howling.	0.939	0.333	13.777	<0.001*
**Miscellaneous**				
Chews inappropriate objects.	1.300	0.253	27.21	<0.001*
Licks him/herself excessively.	0.005	0.945	0.222	0.637
‘Mounts’ objects, furniture, or people.	2.997	0.083	1.086	0.297
Pulls excessively hard when on the leash.	24.107	<0.001*	10.574	0.001*
Rolls in animal droppings or other ‘smelly’ substances.	15.355	<0.001*	58.347	<0.001*

* Significant p values at the level 0.05 after Holm Bonferroni correction

Attempts to fetch objects decreased for dogs with higher PLGH [(OR = 0.993, Uncorrected 95% CI = 0.991–0.995)) and in dogs with a higher AAS [(OR = 0.997, Uncorrected 95% CI = 0.997- 0.998)) while a reduction in recall when off leash was associated with higher PLGH ((OR = 0.997, Uncorrected 95% CI = 0.995–0.999), but not with a higher AAS.

Finally, there was an increase in the likelihood of dogs with higher PLGH female dogs howling [(OR = 1.004, Uncorrected 95% Confidence interval = 1.002–1.006), and an increase in the likelihood of dogs with a higher PLGH chewing inappropriate objects (OR = 1.004, Uncorrected 95% Confidence interval = 1.003–1.006).

## Discussion

### PLGH correlated with behavioural changes

As in the associated study on neutered male dogs [31], we found that female dogs with less exposure to their natural gonadal hormones (decreased PLGH) showed greater incidence of several fear/anxiety, aggressive, and excitable behaviours than entire female dogs in contexts such as being barked or growled at by an unfamiliar dog, when the doorbell rings or an unfamiliar person visits the home, and when approached by an unfamiliar male dog. This is consistent with Balogh and colleagues, who found that owners of spayed dogs described more frequent and/or more intense fear reaction in their dogs in response to loud noises, unfamiliar objects approaching on or near the sidewalk, or if they were approached by unknown dogs barking, growling or jumping [66]. One previous study on female German shepherd dogs reported an increase in owner-reported emotional reactivity after ovariohysterectomy [65]. With more owner-reported data supporting this finding reported here across multiple breeds and countries, there is a clear and pressing need for longitudinal studies (e.g. using data from VetCompass Australia [78] and doglogbook [79]) that can address possible causality. It is also possible that spaying reduces aggression in some contexts. For example, aggression is seen in cases of pseudopregnancy, which were more common in intact than spayed dogs [48].

Howling when left alone and chewing inappropriate objects were the only behaviours reported less frequently with decreasing lifetime exposure to gonadal hormones. Howling was also reported more frequently in low PLGH male dogs [31]. Howling when left alone has been recorded in association with separation-related distress (SRD) [80–82], although this behaviour was not limited to dogs diagnosed with SRD in one study [83]. Furthermore, it’s possible howling signifies anxiety related to other factors such as confinement, and it is not possible to investigate the function of the howling recorded in C-BARQ responses. A reduction in howling may signify improved welfare if howling is associated with any form of anxiety or distress. Reduced howling may also be perceived as an advantage to owners, particularly those living in high-density city living where excessive vocalisation may lead to complaints from neighbours.

Howling emerged in this study as more commonly reported in female dogs with increasing PLGH. Howling is poorly studied in dogs and the function is not well understood, but due to this association with separation, it is thought it may be a vestigial behaviour to make social contact with absent members of the social group, which is a secondary function of this behaviour in wolves [84]. We posited that communication to attract potential mates may be the task of male members of the species [31], but the current results indicate that this form of communication is present in both sexes and is more commonly reported in dogs with increasing PLGH regardless of sex. It is unknown if reproductive status facilitates social bonding, and these are the first studies to the authors’ knowledge that hint at a possible difference in social bonding between entire and gonadectomised dogs.

Chewing in high PLGH female dogs was not mirrored in the related study on male dogs, where inappropriate chewing was not significantly different between high and low PLGH male dogs [12]. Dogs appear to be motivated to chew, and it is thought to assist in teeth and gum health [85], however, chewing may also be a symptom of distress in kennelled dogs [86]. Chewing inappropriate items may be perceived as a problem behaviour by owners, and–like howling–may lead to breakdown of the human-animal bond [83]. A reduction in this behaviour may be perceived as an advantage by owners.

Chewing non-food and non-toy items has been reduced in laboratory beagles by providing human socialization sessions and environmental enrichment [87]. However, the same study recorded an increase in inappropriate chewing after play sessions with other dogs, suggesting that this behaviour may be more closely associated with arousal than with social interactions. It is therefore difficult to explain why this pattern has been detected in female dogs but not male dogs. Further research should seek confirmation of this pattern in other populations of dogs.

### PLGH vs AAS

Some results differed between PLGH and AAS. PLGH can be considered a measure of how much of the dog’s life has been under the influence of unaltered levels of gonadal hormones. For example, a dog that is gonadectomised at 8 months and has C-BARQ filled out for them soon after will be less exposed to gonadal hormones overall than a dog gonadectomised at 5 years that has C-BARQ filled out for them 3 years later, but will have a higher PLGH. A reduction in dogs coming when called when off leash was recorded in dogs with increasing PLGH, but this relationship did not exist for dogs with higher AAS. Also the increase in reporting dogs howling or chewing with increased PLGH did not hold for AAS. The likelihood of aggressive behaviours towards other dogs increased with decreasing PLGH, but there was no significant effect of AAS. This result may indicate behaviours that are more strongly influenced by hormones than by age–and by extension, learning. For male dogs, age at castration (AAC) has been proposed as a more useful metric to understand behaviours that develop with sexual maturity and are resistant to extinction [31]. There were no behaviours in the current analysis of female dogs that increased or decreased with AAS but not PLGH. This may be a factor of the age at which female dogs are typically gonadectomised, or it is possible that behaviours that develop under the influence of hormones in female dogs are also maintained to some degree by a hormonal effect.

### Limitations

Studies using data from the C-BARQ are sometimes challenged because they may reflect some owner bias but the C-BARQ results have been independently validated by comparison with the results of observational assessments [88–90]. That said, we acknowledge that respondents generally need to seek out the C-BARQ to participate in the survey and, as such, are perhaps more likely to include skilled owners who may also have different views about the timing of gonadectomy when compared with companion dog owners as a whole and may manage entire dogs more successfully than less responsible owners, who may be less attentive to their dog’s behaviour. We also acknowledge that the current study has not dealt with potential breed differences in the effects of spaying before and after puberty. This would require validated information on the age at puberty of each dog described. The chief merit of such breed-specific studies is to unpack the role of breed differences, such as in fearfulness, excessive vocalisation and aggressive behaviours.

Despite the large-scale nature of this study, we cannot determine a cause-effect relationship between gonadectomy and behaviour, as there are multiple confounding factors including but not limited to age, breed, general health condition, diet, lifestyle, socialisation and household dynamics. For example, while a number of studies showed an increased risk of some cancers in spayed dogs, this could be partly explained by studies showing that spayed dogs live longer, and cancer risk increases with age [52]. But what remains unexplored is the potential difference in care [67] or even owner attachment style [91] experienced by neutered and unneutered dogs.

Furthermore, factors including genetics, epigenetics, proteomics and metabolomics are not taken into account in this and the majority of currently published studies investigating links between gonadectomy and physical or behavioural parameters [33]. This and other studies investigating the impact of neutering, whether on physical health or behaviour, are retrospective, observational studies with significant limitations in identifying causal relationships [92]. One of the challenges is that the true incidence of most dog health and behaviour problems is not known. Ideally, large-scale prospective case control cohort studies may clarify the effects of gonadectomy in female dogs, but such studies may be cost-prohibitive and are not without ethical challenges. Consistency between studies identifying specific associations between spaying and behaviour will strengthen, although not confirm definitively, the hypothesis that there is a causal link [67].

### Weighing costs versus benefits of spaying

Significant ethical challenges complicate generic recommendations for gonadectomy, given continued canine overpopulation in many parts of the world [33]. The challenge is that the benefits of neutering outweigh the risks at the level of the population, but this is more nuanced at the level of the individual dog. Nonetheless, two reviews concluded that, on balance, the health and welfare of female dogs over the long term is more likely to be enhanced if those dogs are spayed [67,93].

The decision whether or not to gonadectomise is closely followed by the decision as to when to perform the surgery. For example, maximal reduction of mammary tumour risk favours earlier gonadectomy, whereas maximal reduction of the risk of atrophic vaginitis and urethral sphincter mechanism incompetence favours later gonadectomy.

Responsible pet ownership does not end with having one’s pet gonadectomised. Rearing dogs and managing them in ways that meet their behavioural needs and enrich the bonds they share with their owners must be given priority as a form of preventative care. The challenges that owners face and the role of unwanted behaviours in jeopardising the human-dog bond should not be underestimated by simple, broad-scale policies.

### Recommendations

- There is a need for prospective, long-term studies across a range of breeds.
- There is a need to determine how recommendations concerning the timing of spaying could incorporate factors including the urgency of the need to prevent mating, the risk of future owners overlooking the need to spay, the circumstances under which the bitch is managed, the breed’s predisposition to disorders and the training expertise of the owner.
- In addition to discussing the potential risks and benefits of surgical neutering, veterinarians should discuss other factors including training, diet, lifestyle, environment and preventative care

## Conclusions

This study corroborates results from the related study on behaviour in relation to exposure to gonadal hormones in male dogs, showing a similar increased likelihood of owners reporting fearful and aggressive behaviour in their dogs with decreased exposure to gonadal hormones. Although further exploration of the relationships among peri-pubertal brain development, subsequent learning and eventual behaviour are merited, the current findings suggest that entire female dogs showed a higher likelihood of howling than gonadectomized female dogs, a relationship that was also reported in male dogs. Unlike male dogs, female dogs with longer exposure to gonadal hormones were more likely to be reported as chewers of inappropriate objects.

These findings may be helpful to veterinarians, behaviourists and trainers advising dog owners about behavioural management strategies prior to and after spaying.

Ultimately the findings echo previously voiced concerns about making blanket recommendations regarding the neutering of dogs, and the potential for long-term unintended harms [93]. In addition to discussing the potential risks and benefits of surgical neutering, veterinarians should discuss other factors including training, diet, lifestyle, environment and preventative care [94].

Aggression and fearful behaviour in dogs can emerge through a large variety of processes, and the importance of learning and genetic influences cannot be overstated. It may be alluring for dog owners and breeders to blame gonadectomy for behaviour problems that could be addressed through training and standard behaviour modification procedures, and thus absolve themselves of responsibility and fail to address them. This is a critical issue for dog owners who may not currently be adequately advised by veterinary professionals on the best course of action for their individual dog. The lack of experimental evidence combined with great interest from the dog-owning public may result in the rise of strong beliefs regarding what is best for dogs as a whole that are resistant to change through exposure to new evidence. Strong and rigid beliefs may hamper an individual approach to dog welfare and management

## Supporting information

S1 Table Numbers of dogs of the 27 breeds and crosses tested in the current study. (DOCX)

S2 Table C-BARQ attributes showing percentage differences of high C-BARQ item scores between entire and spayed female dogs. A negative valence in the % difference column indicates that entire dogs show high levels of the behaviour less frequently than spayed dogs.
(DOCX)

S1 Fig The distribution of lifetime exposure to gonadal hormones in the spayed female dogs (n = 8981) in the current study. (TIF)

## References

[1] Animal Medicines Australia. Pet Ownership in Australia 2016 Research N, Communications N, editors. Newgate Communications; 2016 p. 66.

[2] Pet Food Manufacturer's Association. Dog population 2018. PFMA, editor. Pet Population 2018. 2018.

[3] American Pet Products Association. 2017–2018 APPA National Pet Owners Survey. In: americanpetproducts.org [Internet]. APPA; 2018 [cited 2018]. Available: https://americanpetproducts.org/pubs_survey.asp

[4] Masters AM, McGreevy PD. Dogkeeping practices as reported by readers of an Australian dog enthusiast magazine. AUST VET J. Wiley Online Library; 2008;86: 18–25. 10.1111/j.1751-0813.2007.00248.x 18271818

[5] Beaver BV. Owner complaints about canine behavior. J AM VET MED ASSOC. 1994;204: 1953–1955. 8077144

[6] Hart BL, Eckstein RA. The role of gonadal hormones in the occurrence of objectionable behaviours in dogs and cats. APPL ANIM BEHAV SCI. Elsevier; 1997;52: 331–344.

[7] Lockwood R. The ethology and epidemiology of canine aggression The domestic dog: its evolution, behaviour, and interactions with people. Cambridge, New York: Cambridge University Press; 2017 pp. 160–181.

[8] Roll A, Unshelm J. Aggressive conflicts amongst dogs and factors affecting them. APPL ANIM BEHAV SCI. 1997;52: 229–242.

[9] Line S, Voith V. Dominance aggression of dogs towards people: behavior profile and response to treatment. Applied Animal Behaviour Science. 1986;16: 77–83.

[10] Svartberg K, Forkman B. Personality traits in the domestic dog (*Canis familiaris*). APPL ANIM BEHAV SCI. 2002;79: 133–155.

[11] Starling MJ, Branson N, Thomson PC, McGreevy PD. Age, sex and reproductive status affect boldness in dogs. VET J. Elsevier Ltd; 2013;197: 868–872. 10.1016/j.tvjl.2013.05.019 23778256

[12] Blackshaw JK. An overview of types of aggressive behaviour in dogs and methods of treatment. APPL ANIM BEHAV SCI. Elsevier; 1991;30: 351–361.

[13] Podberscek AL, Serpell JA. Environmental influences on the expression of aggressive behaviour in English Cocker Spaniels. APPL ANIM BEHAV SCI. Elsevier; 1997;52: 215–227.

[14] Guy NC, Luescher UA, Dohoo SE, Spangler E, Miller JB, Dohoo IR, et al A case series of biting dogs: characteristics of the dogs, their behaviour, and their victims. APPL ANIM BEHAV SCI. Elsevier; 2001;74: 43–57.

[15] Spain CV, Scarlett JM, Houpt KA. Long-term risks and benefits of early-age gonadectomy in dogs. Journal of the American Veterinary Medical Association. 2004;224: 380–387. 10.2460/javma.2004.224.380 14765797

[16] Reisner IR, Houpt KA, Shofer FS. National survey of owner-directed aggression in English Springer Spaniels. Journal of the American Veterinary Medical Association. 2005;227: 1594–1603. 10.2460/javma.2005.227.1594 16313036

[17] Maarschalkerweerd RJ, Endenburg N, Kirpensteijn J, Knol BW. Influence of orchiectomy on canine behaviour. VET REC. 2nd ed. 1997;140: 617–619. 10.1136/vr.140.24.617 9228691

[18] McGreevy P, Masters A. Risk factors for separation-related distress and feed-related aggression in dogs: Additional findings from a survey of Australian dog owners. APPL ANIM BEHAV SCI. 2008;109: 320–328. 10.1016/j.applanim.2007.04.001

[19] Trevejo R, Yang M, Lund EM. Epidemiology of surgical castration of dogs and cats in the United States. J AM VET MED ASSOC. 2011;238: 898–904. 10.2460/javma.238.7.898 21453178

[20] O'Neill DG, Church DB, McGreevy PD, Thomson PC, Brodbelt DC. Prevalence of Disorders Recorded in Dogs Attending Primary-Care Veterinary Practices in England. Rosenfeld CS, editor. PLOS ONE. 2014;9: e90501–16. 10.1371/journal.pone.0090501 24594665 PMC3942437

[21] Sánchez-Vizcaíno F, Noble P-JM, Jones PH, Menacere T, Buchan I, Reynolds S, et al Demographics of dogs, cats, and rabbits attending veterinary practices in Great Britain as recorded in their electronic health records. BMC VET RES. BioMed Central; 2017;13: 218 10.1186/s12917-017-1138-9 28693574 PMC5504643

[22] Kubinyi E, Turcsán B, Miklósi Á. Dog and owner demographic characteristics and dog personality trait associations. BEHAV PROCESS. 2009;81: 392–401. 10.1016/j.beproc.2009.04.004 19520239

[23] Howe LM. Current perspectives on the optimal age to spay/castrate dogs and cats. Veterinary medicine (Auckland, NZ). 2015 ed. 2015;6: 171–180. 10.2147/vmrr.S53264 30101104 PMC6070019

[24] Farhoody P, Mallawaarachchi I, Tarwater PM, Serpell JA, Duffy DL, Zink C. Aggression toward Familiar People, Strangers, and Conspecifics in Gonadectomized and Intact Dogs. FRONT VET SCI. 2018;5: 239–13. 10.3389/fvets.2018.00239 29536014 PMC5834763

[25] Root Kustritz MV. Pros, Cons, and Techniques of Pediatric Neutering. VET CLIN NORTH AM SMALL ANIM PRACT. 2014;44: 221–233. 10.1016/j.cvsm.2013.10.002 24580988

[26] Marston LC, Bennett PC, Coleman GJ. What Happens to Shelter Dogs? An Analysis of Data for 1 Year From Three Australian Shelters. J APPL ANIM WELF SCI. 2004;7: 27–47. 10.1207/s15327604jaws0701_2 15066769

[27] Patronek GJ, Glickman LT, Moyer MR. Population dynamics and the risk of euthanasia for dogs in an animal shelter. ANTHROZOOS. 1995;8: 31–43.

[28] Kass PH, New JC Jr., Scarlett JM, Salman MD. Understanding Animal Companion Surplus in the United States: Relinquishment of Nonadoptables to Animal Shelters for Euthanasia. J APPL ANIM WELF SCI. 2001;4: 237–248. 10.1207/S15327604JAWS0404_01

[29] McGreevy PD, Bennett PC. Challenges and paradoxes in the companion-animal niche. ANIMAL WELFARE-POTTERS BAR. Universities Federation for Animal Welfare; 2010;19: 11–16.

[30] Boyd C, Jarvis S, McGreevy P, Heath S, Church D, Brodbelt D, et al Mortality resulting from undesirable behaviours in dogs aged under three years attending primary-care veterinary practices in England. Animal Welfare. 2018;27: 251–262. 10.7120/09627286.27.3.251

[31] McGreevy PD, Wilson B, Starling MJ, Serpell JA. Behavioural risks in male dogs with minimal lifetime exposure to gonadal hormones may complicate population-control benefits of desexing. Yildirim A, editor. PLOS ONE. 2018;13: e0196284–14. 10.1371/journal.pone.0196284 29718954 PMC5931473

[32] O’Neill DG, Church DB, McGreevy PD, Thomson PC, Brodbelt DC. Longevity and mortality of owned dogs in England. VET J. Elsevier Ltd; 2013;198: 638–643. 10.1016/j.tvjl.2013.09.020 24206631

[33] Smith A. The Role of Neutering in Cancer Development. VET CLIN NORTH AM SMALL ANIM PRACT. 2014;44: 965–975. 10.1016/j.cvsm.2014.06.003 25174910

[34] Reichler IM. Gonadectomy in Cats and Dogs: A Review of Risks and Benefits. REPROD DOMEST ANIM. Wiley/Blackwell (10.1111); 2009;44: 29–35. 10.1111/j.1439-0531.2009.01437.x 19754532

[35] Hagman R. Canine pyometra: What is new? REPROD DOMEST ANIM. 2017;52: 288–292. 10.1111/rda.12843 27807901

[36] Jitpean S, Hagman R, Ström Holst B, Höglund OV, Pettersson A, Egenvall A. Breed Variations in the Incidence of Pyometra and Mammary Tumours in Swedish Dogs. REPROD DOMEST ANIM. Wiley/Blackwell (10.1111); 2012;47: 347–350. 10.1111/rda.12103 23279535

[37] Hagman R. Pyometra in Small Animals. VET CLIN NORTH AM SMALL ANIM PRACT. 2018;48: 639–661. 10.1016/j.cvsm.2018.03.001 29933767

[38] Egenvall A, Hagman R, Bonnett BN, Hedhammar Å, Olson P, Lagerstedt A-S. Breed Risk of Pyometra in Insured Dogs in Sweden. J VET INTERN MED. Wiley/Blackwell (10.1111); 2001;15: 530–538. 10.1892/0891-6640(2001)015<0530:bropii>2.3.co;2 11817057

[39] Gibson A, Dean R, Yates D, Stavisky J. A retrospective study of pyometra at five RSPCA hospitals in the UK: 1728 cases from 2006 to 2011. VET REC. 2013;173: 396 10.1136/vr.101514 24114733 PMC3812855

[40] Kyles AE, Douglass JP, Rottman JB. Pyelonephritis following inadvertent excision of the ureter during ovariohysterectomy in a bitch. VET REC. 1996;139: 471–472. 10.1136/vr.139.19.471 8938968

[41] Dow C. The Cystic Hyperplasia-Pyometra Complex in the Bitch. Journal of Comparative Pathology and Therapeutics. Elsevier; 1959;69: 237–IN18. 10.1016/S0368-1742(59)80023-0 13817874

[42] van Goethem B, SCHAEFERS-OKKENS A, KIRPENSTEIJN J. Making a rational choice between ovariectomy and ovariohysterectomy in the dog: a discussion of the benefits of either technique. Veterinary Surgery. 2006;35: 136–143. 10.1111/j.1532-950X.2006.00124.x 16472293

[43] Schneider R, Dorn CR, Taylor DO. Factors influencing canine mammary cancer development and postsurgical survival. J NATL CANCER INST. 1969;43: 1249–1261. 4319248

[44] Beauvais W, Cardwell JM, Brodbelt DC. The effect of neutering on the risk of mammary tumours in dogs—a systematic review. J SMALL ANIM PRACT. 2012;53: 314–322. 10.1111/j.1748-5827.2011.01220.x 22647210

[45] Byron JK, Taylor KH, Phillips GS, Stahl MS. Urethral Sphincter Mechanism Incompetence in 163 Neutered Female Dogs: Diagnosis, Treatment, and Relationship of Weight and Age at Neuter to Development of Disease. J VET INTERN MED. Hoboken: John Wiley and Sons Inc; 2017;31: 442–448. 10.1111/jvim.14678 28256023 PMC5354041

[46] Johnston SD, Root Kustritz MV, Olson PNS. Canine and Feline Theriogenology Canine and Feline Theriogenology. Philadelphia: WB Saunders; 2001 pp. 80–87.

[47] Root Kustritz MV. Effects of Surgical Sterilization on Canine and Feline Health and on Society. REPROD DOMEST ANIM. Wiley/Blackwell (10.1111); 2012;47: 214–222. 10.1111/j.1439-0531.2012.02078.x 22827373

[48] Root AL, Parkin TD, Hutchison P, Warnes C, Yam PS. Canine pseudopregnancy: an evaluation of prevalence and current treatment protocols in the UK. BMC VET RES. 2018;14: 170 10.1186/s12917-018-1493-1 29793494 PMC5968611

[49] McKenzie R. Species and hybrid cultivars ofLilium(lilies). In: McKenzie R, editor. Australia's Poisonous Plants, Fungi and Cyanobacteria. CSIRO Publishing; 2012.

[50] Jitpean S, Ström-Holst B, Emanuelson U, Höglund OV, Pettersson A, Alneryd-Bull C, et al Outcome of pyometra in female dogs and predictors of peritonitis and prolonged postoperative hospitalization in surgically treated cases. BMC VET RES. BioMed Central; 2014;10: 6–6. 10.1186/1746-6148-10-6 24393406 PMC3892096

[51] Hart BL, Hart LA, Thigpen AP, Willits NH. Neutering of German Shepherd Dogs: associated joint disorders, cancers and urinary incontinence. Veterinary Medicine and Science. Wiley-Blackwell; 2016;2: 191–199. 10.1002/vms3.34 29067194 PMC5645870

[52] Kent MS, Burton JH, Dank G, Bannasch DL, Rebhun RB. Association of cancer-related mortality, age and gonadectomy in golden retriever dogs at a veterinary academic center (1989–2016). PLOS ONE. Public Library of Science; 2018;13: e0192578 10.1371/journal.pone.0192578 29408871 PMC5800597

[53] Adin CA. Complications of Ovariohysterectomy and Orchiectomy in Companion Animals. VET CLIN NORTH AM SMALL ANIM PRACT. 2011;41: 1023–1039. 10.1016/j.cvsm.2011.05.004 21889699

[54] DeLay J. Perianesthetic Mortality in Domestic Animals: A Retrospective Study of Postmortem Lesions and Review of Autopsy Procedures. VET PATHOL. SAGE Publications Inc; 2016;53: 1078–1086. 10.1177/0300985816655853 27371539

[55] Levy JK, Bard KM, Tucker SJ, Diskant PD, Dingman PA. Perioperative mortality in cats and dogs undergoing spay or castration at a high-volume clinic. VET J. British Association of Oral and Maxillofacial Surgeons; 2017;224: 11–15. 10.1016/j.tvjl.2017.05.013 28697869

[56] Hagman R. Pyometra in Small Animals. VET CLIN NORTH AM SMALL ANIM PRACT. 2018;48: 639–661. 10.1016/j.cvsm.2018.03.001 29933767

[57] O’Neill DG, Riddell A, Church DB, Owen L, Brodbelt DC, Hall JL. Urinary incontinence in bitches under primary veterinary care in England: prevalence and risk factors. J SMALL ANIM PRACT. Wiley/Blackwell (10.1111); 2017;58: 685–693. 10.1111/jsap.12731 28881018

[58] Seguin MA, Vaden SL, Altier C, Stone E, Levine JF. Persistent Urinary Tract Infections and Reinfections in 100 Dogs (1989–1999). J VET INTERN MED. Wiley/Blackwell (10.1111); 2008;17: 622–631. 10.1111/j.1939-1676.2003.tb02492.x 14529127

[59] Kustritz MV. Determining the optimal age for gonadectomy of dogs and cats. Journal of the American Veterinary Medical Association. 2007 ed. 2007;231: 1665–1675. 10.2460/javma.231.11.1665 18052800

[60] Belanger JM, Bellumori TP, Bannasch DL, Famula TR, Oberbauer AM. Correlation of neuter status and expression of heritable disorders. CANINE GENET EPIDEMIOL. 2017;4: 6 10.1186/s40575-017-0044-6 28560045 PMC5445488

[61] Torres de la Riva G, Hart BL, Farver TB, Oberbauer AM, Messam LLM, Willits N, et al Neutering Dogs: Effects on Joint Disorders and Cancers in Golden Retrievers. PLOS ONE. Public Library of Science; 2013;8: e55937 10.1371/journal.pone.0055937 23418479 PMC3572183

[62] Bermingham EN, Thomas DG, Cave NJ, Morris PJ, Butterwick RF, German AJ. Energy Requirements of Adult Dogs: A Meta-Analysis. PLOS ONE. Public Library of Science; 2014;9: e109681 10.1371/journal.pone.0109681 25313818 PMC4196927

[63] Overall KL, Beebe AD. Dominance aggression in young female dogs: what does this suggest about the heterogeneity of the disorder. 1997.

[64] Bálint A, Rieger G, Miklósi Á, Pongrácz P. Assessment of owner-directed aggressive behavioural tendencies of dogs in situations of possession and manipulation. R SOC OPEN SCI. 2017;4. 10.1098/rsos.171040 PMC5666282 29134099

[65] Kim HH, Yeon SC, Houpt KA, Lee HC, Chang HH, Lee HJ. Effects of ovariohysterectomy on reactivity in German Shepherd dogs. VET J. 2006;172: 154–159. 10.1016/j.tvjl.2005.02.028 16772140

[66] Balogh O, Borruat N, Andrea Meier A, Hartnack S, Reichler IM. The influence of spaying and its timing relative to the onset of puberty on urinary and general behaviour in Labrador Retrievers. REPROD DOMEST ANIM. Wiley/Blackwell (10.1111); 2018;53: 1184–1190. 10.1111/rda.13225 29974985

[67] McKenzie B. Evaluating the benefits and risks of neutering dogs and cats. CAB Reviews: Perspectives in Agriculture, Veterinary Science, Nutrition and Natural Resources. Wallingford: CABI; 2010;5: 1–18. 10.1079/PAVSNNR20105045

[68] Heidenberger E, Unshelm J. Changes in the behavior of dogs after castration. TIERÄRZTLICHE. 1990;18: 69–75. 2326799

[69] McGreevy PD. A Modern Dog's Life. Randwick, NSW: UNSW Press; 2009.

[70] O'Farrell V, Peachey E. Behavioural effects of ovariohysterectomy on bitches. J SMALL ANIM PRACT. 1990;31: 595–598.

[71] Takeuchi Y, Houpt K, Scarlett J. Evaluation of treatments for separation anxiety in dogs. J AM VET MED ASSOC. 2000;217: 342–345. 10.2460/javma.2000.217.342 10935036

[72] Mongillo P, Scandurra A, D’Aniello B, Marinelli L. Effect of sex and gonadectomy on dogs’ spatial performance. APPL ANIM BEHAV SCI. Elsevier B.V; 2017;191: 84–89. 10.1016/j.applanim.2017.01.017

[73] Scandurra A, Marinelli L, Lõoke M, D’Aniello B, Mongillo P. The effect of age, sex and gonadectomy on dogs’ use of spatial navigation strategies. APPL ANIM BEHAV SCI. Elsevier; 2018;205: 89–97. 10.1016/j.applanim.2018.05.010

[74] Scandurra A, Alterisio A, Di Cosmo A, D’Ambrosio A, D’Aniello B. Ovariectomy Impairs Socio-Cognitive Functions in Dogs. ANIMALS. 2019;9: 58–7. 10.3390/ani9020058 30769794 PMC6406991

[75] Hsu Y, Serpell JA. Development and validation of a questionnaire for measuring behavior and temperament traits in pet dogs. Journal of the American Veterinary Medical Association. 2003;223: 1293–1300. 10.2460/javma.2003.223.1293 14621216

[76] Duffy DL, Serpell JA. Predictive validity of a method for evaluating temperament in young guide and service dogs. APPL ANIM BEHAV SCI. Elsevier B.V; 2012;138: 99–109. 10.1016/j.applanim.2012.02.011

[77] Agresti A. Categorical Data Analysis. Hoboken, New Jersey: John Wiley & Sons, Inc; pp. 282–284.

[78] McGreevy P, Thomson P, Dhand N, Raubenheimer D, Masters S, Mansfield C, et al VetCompass Australia: A National Big Data Collection System for Veterinary Science. ANIMALS. 2017;7: 74–15. 10.3390/ani7100074 28954419 PMC5664033

[79] McGreevy P, Starling M, Payne E, Bennett P. Defining and measuring dogmanship: A new multidisciplinary science to improve understanding of human–dog interactions. VET J. Elsevier Ltd; 2017;229: 1–5. 10.1016/j.tvjl.2017.10.015 29183567

[80] Appleby D, Pluijmakers J. Separation anxiety in dogs: the function of homeostasis in its development and treatment. CLINTECH SMALL AN P. 2004;19: 205–215. 10.1053/j.ctsap.2004.10.002 18371317

[81] Cannas S, Frank D, Minero M, Aspesi A, Benedetti R, Palestrini C. Journal of Veterinary Behavior. J VET BEHAV. Elsevier Ltd; 2014;9: 50–57. 10.1016/j.jveb.2013.12.002

[82] Storengen LM, Boge SCK, Strøm SJ, Løberg G, Lingaas F. A descriptive study of 215 dogs diagnosed with separation anxiety. APPL ANIM BEHAV SCI. Elsevier B.V; 2014;159: 82–89. 10.1016/j.applanim.2014.07.006

[83] Scaglia E, Cannas S, Minero M, Frank D, Bassi A, Palestrini C. Journal of Veterinary Behavior. J VET BEHAV. Elsevier Ltd; 2013;: 1–6. 10.1016/j.jveb.2013.04.065

[84] Taylor AM, Ratcliffe VF, McComb K, Reby D. Auditory communication in domestic dogs: Vocal signalling in the extended social environment of a companion animal In: Kaminski J, Marshall-Pescini S, editors. The Social Dog. 1st ed Oxford, UK; 2014.

[85] Wells D. A review of environmental enrichment for kennelled dogs, *Canis familiaris*. APPL ANIM BEHAV SCI. 2004;85: 307–317.

[86] Rooney N, Gaines S, Hiby E. A practitioner's guide to working dog welfare. J VET BEHAV. Elsevier Inc; 2009;4: 127–134. 10.1016/j.jveb.2008.10.037

[87] Hubrecht R. A comparison of social and environmental enrichment methods for laboratory housed dogs. Applied Animal Behaviour Science. 1993;37: 345–361. 10.1016/0168-1591(93)90123-7

[88] De Meester R, De Bacquer D, Peremans K. A preliminary study on the use of the Socially Acceptable Behavior test as a test for …. J VET BEHAV. 2008.

[89] van der Borg JAM, Beerda B, Ooms M, De Souza AS, Van Hagen M, Kemp B. Evaluation of behaviour testing for human directed aggression in dogs. APPL ANIM BEHAV SCI. Elsevier B.V; 2010;128: 78–90. 10.1016/j.applanim.2010.09.016

[90] De Meester RH, De Bacquer D, Peremans K, Vermeire S, Planta DJ, Coopman F, et al A preliminary study on the use of the Socially Acceptable Behavior test as a test for shyness/confidence in the temperament of dogs. J VET BEHAV. 2008;3: 161–170. 10.1016/j.jveb.2007.10.005

[91] Konok V, Kosztolányi A, Rainer W, Mutschler B, Halsband U, Miklósi Á. Influence of Owners’ Attachment Style and Personality on Their Dogs’ (Canis familiaris) Separation-Related Disorder. PLOS ONE.; 2015;10: e0118375 10.1371/journal.pone.0118375 25706147 PMC4338184

[92] Cockcroft PD, Holmes MA. Handbook of Evidence-based Veterinary Medicine Oxford: Wiley Blackwell; 2003.

[93] Palmer C, Corr S, Sandoe P. Inconvenient desires: should we routinely neuter companion animals? Anthrozoos. 2012;25: S153–S172.

[94] Wolfe TM. The Great Spay/Neuter Debate: Does Early Gonadectomy Prevent or Cause Disease in Dogs? ADV SMALL ANIM MED SURG. 2015;28: 1–3. 10.1016/j.asams.2015.12.001

