# Supplementary material for: Behavioural risks in female dogs with minimal lifetime exposure to gonadal hormones
Source: PLoS One. 2019 Dec 5;14(12):e0223709. doi: 10.1371/journal.pone.0223709 (PMC6894801; doi:10.1371/journal.pone.0223709)
Supplement: S1 Table — (DOCX) [file pone.0223709.s001.docx]

| **Breed reported** | **Number in**  **current study** |
| --- | --- |
| Mixed Breed/Unknown | 2690 |
| RAREBREED | 2623 |
| Labrador Retriever | 553 |
| German Shepherd | 355 |
| Golden Retriever | 286 |
| Border Collie | 235 |
| Australian Shepherd | 184 |
| American Pit Bull Terrier | 162 |
| Rottweiler | 140 |
| Australian Cattle Dog | 121 |
| Poodle (Standard) | 119 |
| Chihuahua | 113 |
| Soft Coated Wheaten Terrier | 106 |
| Shetland Sheepdog | 104 |
| Doberman Pinscher | 103 |
| Boxer | 102 |
| Jack Russell Terrier | 97 |
| Pit Bull mix | 96 |
| Beagle | 87 |
| Newfoundland | 86 |
| Greyhound | 82 |
| Staffordshire Bull Terrier | 75 |
| Cocker Spaniel (American) | 69 |
| Siberian Husky | 68 |
| Miniature Schnauzer | 66 |
| Shiba Inu | 66 |
| American Staffordshire Terrier | 65 |
| Great Dane | 64 |
| Shih Tzu | 64 |
