# Supplementary material for: Behavioural risks in female dogs with minimal lifetime exposure to gonadal hormones
Source: PLoS One. 2019 Dec 5;14(12):e0223709. doi: 10.1371/journal.pone.0223709 (PMC6894801; doi:10.1371/journal.pone.0223709)
Supplement: S2 Table — A negative valence in the % difference column indicates that entire dogs show high levels of the behaviour less frequently than spayed dogs. (DOCX) [file pone.0223709.s002.docx]

| **Percentage  Difference between  entire dogs and  castrated dogs** | C-BARQ section | **Detail** |
| --- | --- | --- |
| **-8.096** | **Trainability** | When off the leash, returns immediately when called. |
| **5.474** | **Trainability** | Easily distracted by interesting sights, sounds or smells. |
| **-5.776** | **Trainability** | Will ‘fetch’ or attempt to fetch sticks, balls, or objects. |
| **-7.098** | **Aggression** | When mailmen or other delivery workers approach your home. |
| **-5.433** | **Aggression** | When approached directly by an unfamiliar **male** dog while being walked/exercised on a leash. |
| **-6.208** | **Aggression** | When barked, growled, or lunged at by another (unfamiliar) dog. |
| **-11.507** | **Fear and Anxiety** | In response to sudden or loud noises (e.g. vacuum cleaner, car backfire, road drills, objects being dropped, etc.). |
| **-10.87** | **Fear and Anxiety** | When examined/treated by a veterinarian |
| **-12.213** | **Fear and Anxiety** | During thunderstorms, firework displays, or similar events. |
| **-5.882** | **Fear and Anxiety** | When first exposed to unfamiliar situations (e.g. first car trip, first time in elevator, first visit to veterinarian, etc.) |
| **-6.82** | **Fear and Anxiety** | In response to wind or wind-blown objects. |
| **--11.878** | **Fear and Anxiety** | When having nails clipped by a household member |
| **-8.63** | **Fear and Anxiety** | When groomed or bathed by a household member. |
| **-5.083** | **Excitability** | When having his/her feet toweled by a member of the household. |
| **-6.534** | **Fear and Anxiety** | When barked, growled, or lunged at by an unfamiliar dog. |
| **5.408** | **Separation related behaviour** | Howling. |
| **-5.468** | **Excitability** | When doorbell rings. |
| **-7.227** | **Excitability** | Just before being taken on a car trip. |
| **-6.899** | **Miscellaneous** | Rolls in animal droppings or other ‘smelly’ substances. |
| **9.672** | **Miscellaneous** | Chews inappropriate objects. |
| **-6.351** | **Miscellaneous** | ‘Mounts’ objects, furniture, or people. |
| **-5.326** | **Miscellaneous** | Pulls excessively hard when on the leash. |
| **-8.403** | **Miscellaneous** | Licks him/herself excessively. |
